# Two Cases of Imperforate Hymen

**Published:** 1846-11

**Authors:** 


*Two cases of imperforate Hymen.* By MM. de Bal and Kluyskens.—*Case* 1.—A girl, aged 18, consulted M. de Bal in February, 1844, for symptoms referred to painful menstruations, and again applied to him the following May. She had then grown thin, and suffered intolerable pain in the kidneys and left hypogastrium; the womb, felt through the abdominal parietes, had the size and consistence it presents at the sixth month of pregnancy. There was continued fever, obstinate vomiting, and the countenance was of a pale yellow colour. These symptoms had at first recurred periodically every fifth week, then ever fourth and third week, but had been continued for the last three weeks. On examination M. de Bal found that the vagina was totally absent, and felt a hard, fibrous, voluminous body through the rectum, not very moveable, but moving with the uterus, and giving the sensation of the presentation of the head of a full grown fœtus. With the finger in the rectum and a catheter in the bladder, M. de Bal ascertained that the distance between the most depending point of the tumour and the point where the orifice of the vagina should exist was two-and-a-half inches, and that to the depth of an inch and-a-half there existed between the bladder and the rectum merely a fibrous cord from one-and-a-half to two lines thick.

On the 23rd of May the bladder and rectum having been emptied, a catheter was passed into the bladder, and M. de Bal introduced his finger into the rectum. A transverse incision was than made between the anus and urethra with a bistoury guarded with lint except at the point, and carried in the direction of the fibrous cord to the depth of one inch four lines, and advancing with suitable precaution, a point was attained where there was a greater interval between the rectum and the bladder. A common trocar was then plunged into the dense tumour whose parietes were three or four lines thick. On withdrawing the stilet a pint and a-half of thick viscid inodorous fluid, resembling well boiled syrup, escaped. The tumour diminished on the instant and descended at least two inches. The cavity was washed out with injections of tepid water, and a gum-elastic canula was left in the wound. Every attempt to dilate the wound failed, an invincible resistance having been experienced especially at its upper third. The patient recovered perfectly, and has menstruated regularly since the operation.

*Case* 2.—A, B. had not menstruated at the age of 18. For the last fifteen months has experienced pains resembling colic, which first recurred at long intervals, then every month, every fortnight, and finally, every day. The abdomen became swollen, the pulse were small, frequent, and she was affected with borborygmata and hysteric symptoms. After having in vain tried several remedies, the patient, pallid and exhausted with suffering, consulted M. Verbeech, who discovered in the abdomen, which was as large as at the full term of pregnancy, a hard smooth tumour ascending to the umbilicus. The valve was well formed, but there was no trace of the vaginal orifice. With a catheter in the bladder and the finger in the rectum, a slender cord alone was found to intervene between those two organs. As death seemed inevitable unless relief was obtained, M. Kluyskens determined to attempt creating an artificial passage. With similar precautions to those taken in the foregoing case, an incision was carried to the depth of three inches, when, on passing in the finger, no tumour could be felt. The incision was carried an inch deeper, when a tumour, supposed to be the womb, was felt, but the os uteri could not be distinguished. The position of the tumour having been well ascertained, a free incision was made into it with a sharp-pointed bistoury guarded with lint to near the point. Upwards of five pounds of viscid blood then escaped, and the tumefaction of the abdomen subsided. Everything went favourably till the tenth day, when inflammatory symptoms set in which caused metritis to be apprehended, but they yielded to antiphlogistics. A gum-elastic canula was introduced from time to time, and in five weeks the patient had perfectly recovered.

Several years have elapsed since the performance of this operation, and the artificial passage has remained permanently open ; the patient, who has married, but has no children, enjoys good health, and has menstruated regularly and without pain, but in the intervals is subject to copious leucorrhœa.—*Dub. Med. Press, from Gaz. Med. de Paris.*

